# Inflammation contributes to NKX3.1 loss and augments DNA damage but does not alter the DNA damage response via increased SIRT1 expression

**DOI:** 10.1186/s12950-015-0057-4

**Published:** 2015-02-15

**Authors:** Bilge Debelec-Butuner, Nursah Ertunc, Kemal Sami Korkmaz

**Affiliations:** Department of Bioengineering, Cancer Biology Laboratory, Faculty of Engineering, Ege University, Bornova, 35100 Izmir, Turkey; Department of Pharmaceutical Biotechnology, Faculty of Pharmacy, Ege University, Bornova, Izmir, 35100 Turkey

**Keywords:** ROS, NKX3.1, Inflammatory microenvironment, Prostate tumor, DNA damage

## Abstract

The oxidative stress response is a cellular defense mechanism that protects cells from oxidative damage and cancer development. The exact molecular mechanism by which reactive oxygen species (ROS) contribute to DNA damage and increase genome instability in prostate cancer merits further investigation. Here, we aimed to determine the effects of NKX3.1 loss on antioxidant defense in response to acute and chronic inflammation in an *in vitro* model. Oxidative stress-induced DNA damage resulted in increased H2AX^(S139)^ phosphorylation (a hallmark of DNA damage), along with the degradation of the androgen receptor (AR), p53 and NKX3.1, upon treatment with conditioned medium (CM) obtained from activated macrophages or H_2_O_2_. Furthermore, the expression and stability of SIRT1 were increased by CM treatment but not by H_2_O_2_ treatment, although the level of ATM^(S1981)^ phosphorylation was not changed compared with controls. Moreover, the deregulated antioxidant response resulted in upregulation of the pro-oxidant QSCN6 and the antioxidant GPX2 and downregulation of the antioxidant GPX3 after CM treatment. Consistently, the intracellular ROS level increased after chronic treatment, leading to a dose-dependent increase in the ability of LNCaP cells to tolerate oxidative damage. These data suggest that the inflammatory microenvironment is a major factor contributing to DNA damage and the deregulation of the oxidative stress response, which may be the underlying cause of the increased genetic heterogeneity during prostate tumor progression.

## Introduction

Oxidative stress contributes to the initiation, promotion and progression of carcinogenesis. Excessive levels of reactive oxygen species (ROS) are generated by exposure to oxidative stress and cause sustained DNA damage. DNA damage occurs during the initiation step of carcinogenesis, and most likely results in abnormal gene expression. Because ROS accumulation results in a subsequent failure of signal transduction at the promotion step, the cells undergo a loss of genomic fidelity during the progression step [1]. Notably, ROS are generated in excess amounts during chronic inflammation, and ROS-mediated DNA damage alters the genetic composition. This damage may promote oncogenic transformation, which occurs when genes encoding essential factors involved in DNA repair, apoptosis and cell cycle regulation are affected [2]. Others have shown that ROS contribute to carcinogenesis by activating signaling pathways that regulate cellular proliferation, angiogenesis and metastasis [3-5]. ROS serve as secondary messengers for the activation of key transcription factors in response to pro-inflammatory cytokines, and they also regulate the transcription of genes involved in inflammatory responses [6,7]. Additionally, a number of protein kinase pathways, such as the MAPK pathway, are activated by oxidative signals during inflammation and inflammatory diseases. These pathways synergistically contribute to the activation of cytokine release, and combined with the loss of adhesion and the release of angiogenic factors, they may eventually contribute to cellular proliferation, differentiation and tumor progression [7]. ROS play important roles in multiple signal transduction pathways, such as those mediated by TNFα [8] and p53. These two pathways can activate each other, as DNA damage caused by TNFα-induced ROS directly induces cell cycle and/or apoptosis regulation by p53 [9]. In addition to the ability of cells to trigger proliferation in response to sustained ROS production at low levels, excess ROS generation or the accumulation of ROS may induce cell death depending on the ROS concentration and cell type [4]. Although ROS can induce apoptosis or necrosis depending on the oxidative level [3], the appropriate cellular responses to ROS production are critical events and may protect the cell from sustained oxidative damage and support cell survival.

To reduce the oxidative damage caused by ROS, the antioxidant response system is activated through several constitutive and inducible detoxification mechanisms, including the expression of enzymes such as glutathione peroxidases (GPx), catalase, superoxide dismutases (SODs), peroxiredoxins (PRDXs), glutathione-S-transferases (GSTs), NADP (H) quinone oxidoreductase (NQO1), epoxide hydrolase, heme oxygenase (HO-1), UDP-glucuronosyl transferases (UGTs), and gamma-glutamylcysteine synthetase. The upregulated expression of these enzymes exhibits a distinct cellular defense to protect cells from oxidative damage and cancer development [7,10].

In addition to the mechanisms that regulate ROS, cell proliferation is controlled by stress-sensing molecules such as Sirtuin 1 (SIRT1), an NAD-dependent deacetylase that responds to the levels of redox pairs NAD+/NADH and NADP+/NADPH. Under oxidative conditions, SIRT1 deacetylates a number of transcription factors, including p53, NBS1 and FOXO, which subsequently contribute to cellular metabolic responses, such as cell cycle regulation and DNA damage [7,11,12]. Therefore, SIRT1 has been linked to tumor cell survival by deregulating apoptosis and promoting senescence. Particularly, in prostate cancer, the deacetylation of AR by SIRT1 represses androgen-induced AR transcription and contributes to AR-induced tumorigenesis [13].

NKX3.1 is an androgen-regulated gene that encodes a homeobox protein with a tumor suppressor function in prostate cells [14,15]. The AR response is ubiquitous in prostate tumors, and NKX3.1 is upregulated by androgens; in contrast, NKX3.1 loss has been reported in prostate tumors [16]. Furthermore, the functional loss of NKX3.1 expression upon cytokine exposure has been reported in previous studies of the inflammatory microenvironment [17,18], strengthening its tumor suppressor role in prostate carcinogenesis. The pro-inflammatory cytokines TNFα and IL-1β induce the C-terminal phosphorylation of NKX3.1 by casein kinase 2 (CK2), resulting in a shortened half-life [17,18]. Additionally, loss of NKX3.1 expression in pathogen *E. coli* infected prostate lobes in mice has been shown to be correlated with reduced AR expression [19]. It was previously reported that the loss of NKX3.1 expression was related not only to the loss of AR transactivating function [17,18] but also to high ROS level upon cytokine exposure, particularly TNFα [17]. Concurrently, the loss of p53 expression was also observed in the inflammatory microenvironment, promoted the progression of prostate cancer, perhaps correlating with increased oxidative stress. This effect was partially restored by suppressing AKT and MDM2 phosphorylations, leading to p53 degradation [18].

In this study, we aimed to identify the role of cytokine-induced NKX3.1 loss in the deregulation of the antioxidant defense during acute and chronic exposure to both cytokines and ROS. Therefore, the effect of antioxidant treatment on the inflammation- and/or oxidative stress-induced degradation of NKX3.1, AR and p53 was analyzed. Cultures of the prostate cancer cell line LNCaP were exposed to conditioned medium (CM) with adjusted amounts of pro-inflammatory cytokines (TNFα) for 24 h for acute treatment and for 2 weeks for chronic treatment. Cells were also chronically treated with H_2_O_2_ for 2 weeks to compare the effects of pro-inflammatory cytokines and ROS exposure.

## Materials and methods

### Macrophage differentiation and conditioned media (CM) collection

The U937 monocyte cell line was cultured in RPMI 1640 medium including 10% FBS (fetal bovine serum) at 37°C with 5% CO_2_. To achieve macrophage differentiation and cytokine production, cells (8×10^5^) were seeded into 75-cm^2^ culture flasks 2 h prior to treatment. Next, PMA was added at a final concentration of 16 nM for 16 h, and the adherent clusters (differentiated monocytes) were maintained. The cells were washed twice before the addition of 20 ml of fresh medium, and the cells were then allowed to rest for 3 h. Then, lipopolysaccharide (LPS) was added at a final concentration of 10 ng/ml to induce cytokine secretion. The cells were incubated for an additional 24 h, and the supernatant (conditioned medium - CM) was collected and filtered (using a 0.22-μm filter) for further use. To ensure that the CM was cell-free, diluted CM was cultured in an empty flask (25 cm^2^) for one week and analyzed.

### Measurement of cytokines in CM

Before feeding the LNCaP cells with the collected CM (cell-free), the TNFα (Invitrogen, USA) levels were analyzed using an ELISA according to the manufacturer’s instructions. Because cytokine exposure is a major component of the inflammatory microenvironment, the times (0, 2, 4, 6, 12 and 24 h) and doses (62.5, 125, 250 or 500 pg/ml TNFα-containing conditioned medium) for the courses of CM treatments were optimized as reported in our previous study [18]. TNFα was chosen as a measure of the CM concentration, which was adjusted by diluting the CM with normal medium before application to the LNCaP cells. As a result, the concentrations of macrophage-secreted cytokines were adjusted and maintained at picogram levels. In our studies, the effective concentration of TNFα was 400 times less than the concentration of recombinant TNFα (rTNFα) (sigma, UK) reported in other studies [17-19].

### Cell culture and treatments

LNCaP cells were obtained from the American Type Culture Collection (ATCC, Manassas, VA, USA) and were propagated as recommended using RPMI 1640 medium supplemented with 10% FBS, L-glutamine (2 mM), penicillin (100 U/ml) and streptomycin (100 μg/ml) at 37°C with 5% CO_2_. For the acute exposures, the CM (62, 125, and 250 pg/ml of TNFα) treatments were performed for 24 h; for the chronic exposures, the CM treatments continued for 2 weeks, and lower doses (50 and 100 pg/ml of TNFα) were used. TNFα concentrations were adjusted by diluting the CM using RPMI 1640 medium as described previously [18]. A chronic oxidative condition was also induced by treating the cells with 50, 100 or 200 μM H_2_O_2_ for 2 weeks for comparison of the effects of cytokine exposure and oxidative stress.

### Transfections

The *NKX3.1* open reading frame was amplified (using the primers F: GGATCCATGCTCAGGGTTCCGGAGCCG and R: GAATTCGGTTGTCACCTGAGCTGGCATTA) and cloned into the pcDNA4/HisMax-TOPO vector (Invitrogen, USA) according to the manufacturer’s instructions to obtain HM-NKX3.1 and the HM-vector constructs. Then, transfections were performed using the Fugene HD reagent (Roche, Germany) for 24 h. The cells were incubated for an additional 18 to 42 h, as appropriate.

The siNKX3.1 and siAR transfections were performed as recommended by the supplier (Dharmacon). Briefly, 4×10^5^ cells were seeded into 6-cm plates, and the medium was changed (w/o antibiotics). A transfection mix was prepared by adding 6 μl of Dharmafect II (tube 1) and 200 pmol of siNKX3.1, siAR or scrambled siRNA (tube 2) into 94 μl of transfection medium (w/o antibiotics and serum). After incubation for 5 min at RT, the tubes were mixed and incubated for 15 min at RT and then added onto the cells dropwise. The transfected cells were incubated for an additional 24 h before harvesting.

### Antibodies

The following antibodies were purchased from the manufacturers: AR (Millipore, USA), p53, pH2AX^(S139)^ and pATM^(S1981)^ (Abcam, UK), GAPDH (Ambion, UK), β-actin and SIRT1 (Sigma, UK), Caspase-3 (R&D, UK), and β-tubulin (ABM, UK). The NKX3.1 custom antibody was a gift from Prof. Dr. F. Saatcioglu (University of Oslo). The HRP-conjugated anti-mouse and anti-rabbit (Amersham, UK) and the AlexaFluor 488- and 594-conjugated secondary antibodies (Invitrogen, USA) were purchased and used as recommended by the manufacturers.

### DCFH assay

LNCaP cells (8×10^3^) were seeded into 96-well plates, and the transfections were carried out on the following day. Two days later, the cells were incubated with DCFH-DA (2′ 7′- dichlorodihydrofluorescein diacetate, Molecular Probes, 10 μM) for 30 min at 37°C. Next, the treatments were performed following gentle washes using phenol red-free medium. Finally, the fluorescence intensity was measured every 20 min for up to 3 h using a Fluoroscan fluorometer (Thermo Science, USA).

### Protein extraction and western blotting

For protein extraction, LNCaP cells were lysed using a modified RIPA buffer (10 mM Tris-Cl (pH: 8.0), 1% Triton X-100, 0.1% SDS, 0.1% Na deoxycholate, 1 mM EDTA, 1 mM EGTA, 140 mM NaCl) containing protease and phosphatase inhibitors. Then, the concentrations were determined using the BCA assay (Sigma, UK). SDS-PAGE and western blots were performed under standard conditions with 50 μg of protein lysate per lane. The proteins were separated on 10-12% gels and transferred to PVDF membranes (Amersham, UK) using a wet transfer blotter. The PVDF membrane was blocked with 5% dry milk in TBS-T (Tris-Buffered-Saline solution containing 0.1% Tween 20). The primary and secondary antibody incubations were performed in TBS-T containing 0.5% dry milk or 5% BSA at RT for 1 h or at 4°C o/n. The membranes were developed using the ECL prime reagent (Amersham, UK) for 5 min and were photographed using Kodak X-Ray films in a dark room.

### Real-time cell proliferation assay

The Xcelligence proliferation assay platform was used for real-time measurements. Briefly, the LNCaP cells (8×10^3^) were transfected with an HM vector and HM-NKX3.1 (24 h), seeded into 96-well plates (E-plates, Roche GmbH, Germany) and cultured for 24 h. The treatments were performed as described, and the proliferation rate and morphological changes were monitored. Impedance values were collected every 10 min for 48 h.

### cDNA synthesis

Total RNA was isolated from the LNCaP cells using the RNeasy kit (Qiagen, CA, USA), and the yield was calculated using absorbance readings at 260/280 nm. Then, cDNA synthesis was performed using a cDNA synthesis kit (Invitrogen, USA) as recommended by the manufacturer.

### Real time PCR

To study the expression of specific genes, quantitative RT-PCR was performed using a SYBR Green PCR kit and the LC480 PCR system (Roche, Germany). The relative abundance of each transcript was calculated using the comparative cycle threshold (CT) method with GAPDH as an invariant control. The following primers were used: GPX2_F: CAGTCTCAAGTATGTCCGT, GPX2_R: AGGCTCAATGTTGATGGT; GPX3_F: CTTGCACCATTCGGTCT, GPX3_R: CGGACATACTTGAGGGTAG; PRDX6_F: TAGTGTGATGGTCCTTCCAAC, PRDX6_R: AGCGGAGGTATTTCTTGC; QSCN6_F: GAGGCTACGTGCACTACT, QSCN6_R: CTGCAAGGCGAGCATTGA; ENOX2_F: CTGAACGTGAAGCACTG, ENOX2_R: ATCAAGACGGTGCAAGTAG; SOD1_F: TGTACCAGTGCAGGTCC, SOD1_R: GCCAATGATGCAATGGTC; SOD2_F: TGTCCAAGGCTCAGGTT, SOD2_R: CTGAAGGTAGTAAGCGTGC; NKX3.1_F: TCTATCAGCATCTGACAGGTGAA, NKX3.1_R: AGCAGGGTTTGTTATGCATGTAG; SIRT1_F: TGCGGGAATCCAAAGGATAATTCAGTGTC, SIRT1_R: CTTCATCTTTGTCATACTTCATGGCTCTATG; and GAPDH_F: CATTGCCCTCAACGACCACTTT, GAPDH_R: GGTGGTCCAGGGGTCTTACTCC.

### Statistics

Student’s *t* test was applied to determine the statistical significance between pairs where necessary.

## Results and discussion

### NKX3.1, AR, and p53 degradation is restored by LNAC treatment

**Figure 1:**
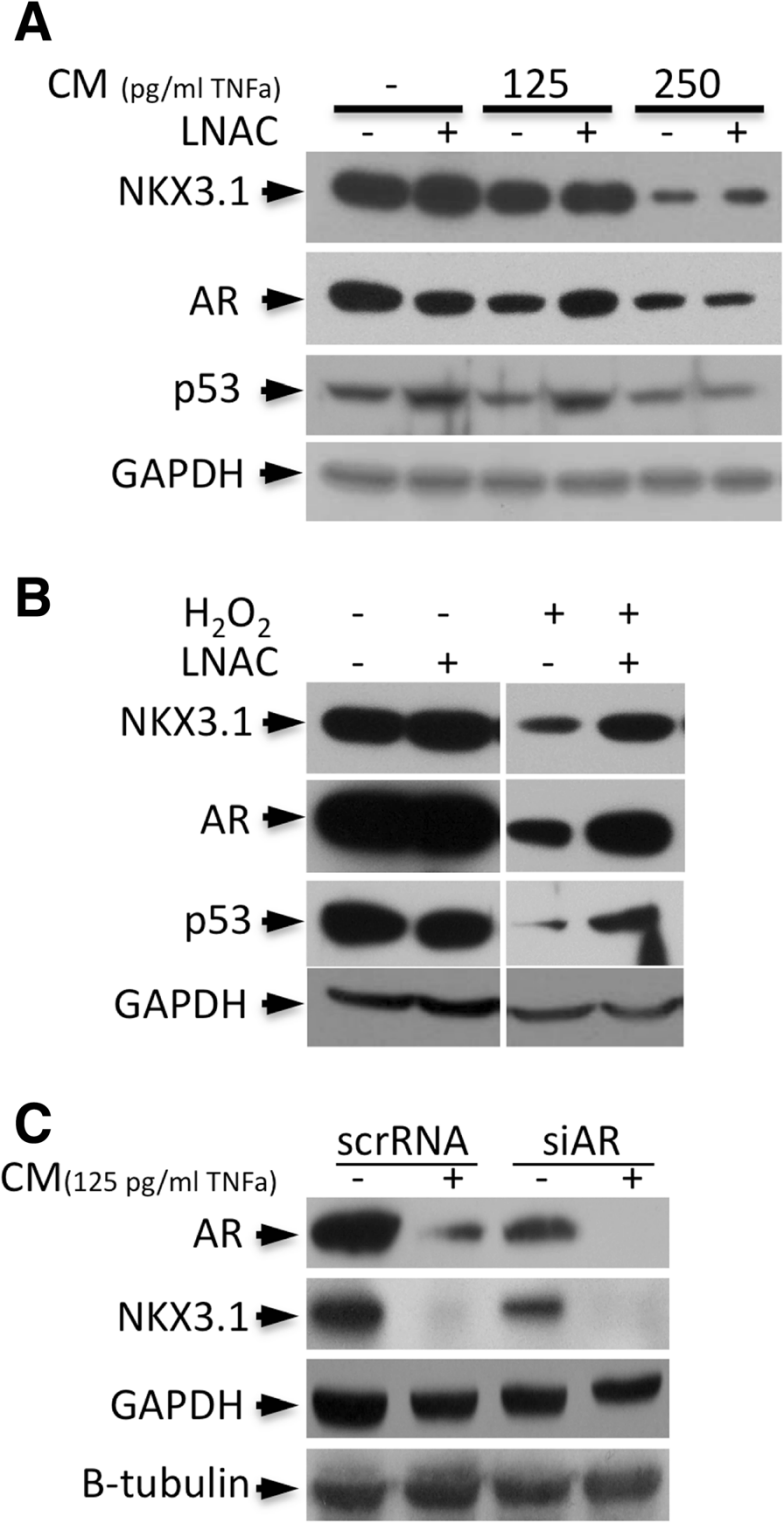
**Antioxidant LNAC treatment restores AR, NKX3.1 and p53 expression levels.** In LNCaP cells, the expression levels that were reduced by treatment with **A**. CM (including 125 and 250 pg/ml of TNFα) and **B**. H_2_O_2_ (250 μM) for 24 h were partially restored back to control expression levels by the antioxidant LNAC (10 mM) treatment. LNAC was applied to the cells 1 h prior to the addition of CM or H_2_O_2_. **C**. AR depletion together with CM treatment reduced the NKX3.1 level. CM: conditioned media.

To determine whether inflammatory cytokine or oxidative exposure is the major factor in ROS-dependent reduction of AR, NKX3.1 and p53 protein levels, LNCaP cells were treated with CM or H_2_O_2_ with or without the antioxidant, N-Acetyl-L-cysteine (LNAC). The CM treatments resulted in the degradation of AR, p53 and NKX3.1 in a dose-dependent manner, which is consistent with our previous studies [18]. Interestingly, LNAC treatment partially restored AR, but not NKX3.1, at low (125 pg/ml of TNFα) concentrations of CM (Figure 1A). Because the antioxidant treatment could not significantly change the expression level of NKX3.1 and AR under inflammatory conditions (CM treatment), cytokine exposure was suggested as the major cause of NKX3.1 and AR depletion. Nevertheless, AR and NKX3.1 degradation in the presence of H_2_O_2_ were almost completely restored back to basal levels by LNAC (Figure 1B), suggesting that there is clear crosstalk between ROS and cytokine signaling. Also, the p53 level was slightly increased by LNAC in the control cells, and it was completely restored back to the basal expression level at lower doses of CM (Figure 1A). Because LNAC also enhanced the p53 level after H_2_O_2_ treatment (Figure 1B), these results implied that p53 degradation under inflammatory conditions was dependent upon the increased ROS levels. Furthermore, we performed AR silencing together with CM treatment in LNCaP cells, and the results showed that the reduced NKX3.1 level was a consequence of the synergetic effects of repression of AR transactivation due to AR depletion and CM-mediated proteasomal degradation (Figure 1C).

### NKX3.1 is required for antioxidant gene expression to limit oxidative damage

**Figure 2:**
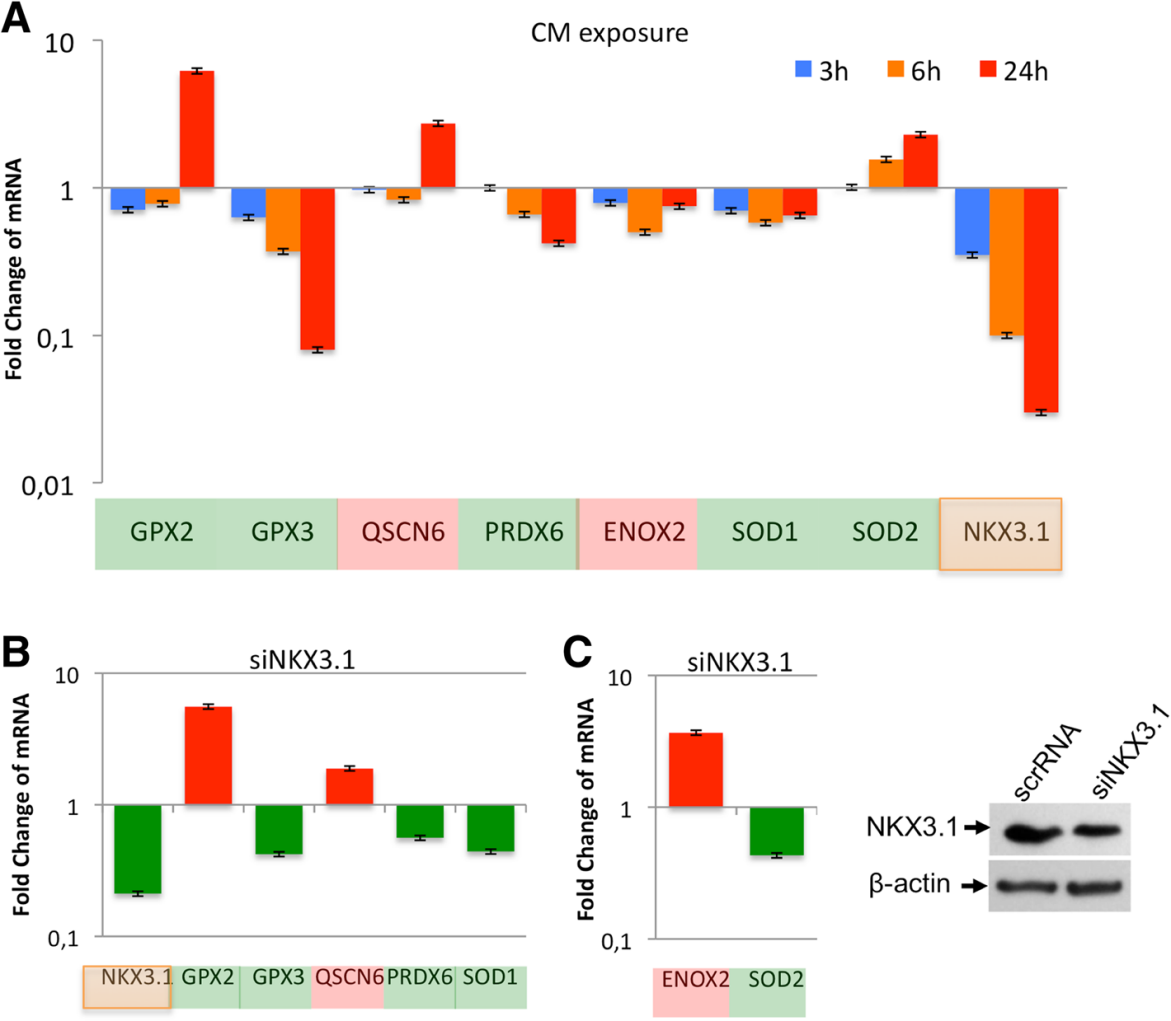
**Depleting NKX3.1 results in alterations in the expression of genes involved in the regulation of oxidative stress. A**. Time course (3, 6 and 24 h) of CM exposure influences gene expression in LNCaP cells. Pink and green colors indicate the gene expression of pro- and anti-oxidant enzymes, respectively. **B**. The changes in gene expression caused by NKX3.1 depletion correlate with the changes that occurred after CM treatment (250 pg/ml of TNFα for 24 h) in LNCaP cells. **C**. Histogram plot demonstrates the genes that underwent changes in expression upon NKX3.1 depletion that did not correlate with CM exposure. Red and green bars indicate upregulation and downregulation, respectively. Western blot confirms the depleted NKX3.1 protein level in comparison to B-actin expression due to NKX3.1 silencing.

NKX3.1 is an important androgen-regulated transcription factor in prostate and testis tissues. Here, CM (250 pg/ml of TNFα) treatments were performed for 3, 6 and 24 h. The results indicate that NKX3.1 loss was correlated with antioxidant responsive gene expression in LNCAP cells. We found that the expression of the pro-oxidants Quiescin Q6 (QSCN6) and Ecto-NOX disulfide thiol exchanger 2 (ENOX2) was increased by 2.7 and 1.3-fold relative to the controls, respectively. The antioxidant glutathione peroxidase-2 (GPX2) was upregulated 6.2-fold, and glutathione peroxidase-3 (GPX3) and peroxiredoxin-6 (PRDX6) were downregulated 12.5- and 2.4-fold, respectively. Further, the antioxidant superoxide dismutase-1 (SOD1) was downregulated 1.5-fold, whereas superoxide dismutase-2 (SOD2) was upregulated 2.3-fold after 24 h of CM treatment (Figure 2A). Because an approximately 30-fold reduction of NKX3.1 was observed upon CM exposure, these data support the finding that the loss of NKX3.1 could be related not only to cytokine exposure [17] but also to the loss of the androgen receptor.

**Figure 3:**
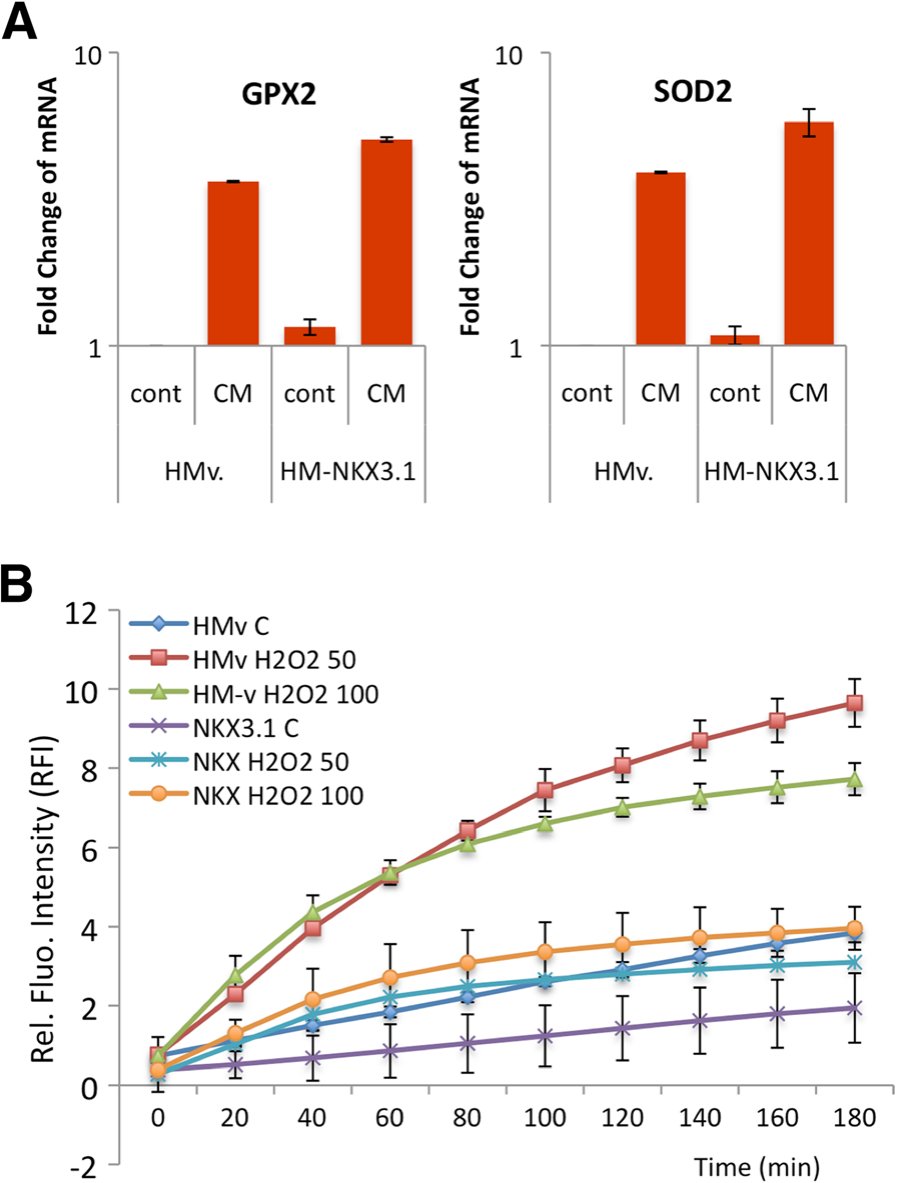
**Overexpression of NKX3.1 leads to the suppression of oxidative stress. A**. The CM (250 pg/ml of TNFα for 24 h)-mediated increase in GPX2 and SOD2 expression is upregulated upon NKX3.1 overexpression in LNCaP cells. **B**. The H_2_O_2_-induced intracellular ROS level is suppressed by NKX3.1. Control and NKX3.1-expressing constructs (pcDNA4-HM) were transfected into the cells 24 h prior to H_2_O_2_ treatment, p < 0.001.

To investigate whether the loss of the oxidative stress response in prostate cancer cells was related to the loss of NKX3.1 expression or androgen signaling during inflammation, NKX3.1 was depleted by transfection with siRNA for 48 h, and the mRNA levels of specific genes were quantified. The analyzed genes were classified into two groups. The first group consisted of the genes whose expression was dose-dependently affected by treatment with CM. This group included GPX2 and QSCN6, which were upregulated by 5.6-fold and 1.9-fold, respectively, and PX3, PRDX6 and SOD1, which were downregulated by 2.4-fold, 2-fold and 2.5-fold, respectively (Figure 2B and C). The second group consisted of the genes whose expression was influenced by NKX3.1 depletion but not by CM treatment (Figure 2B and C). This group included ENOX2, which was upregulated by 3.7-fold compared to the control, and SOD2, which was downregulated by 2.5-fold. NKX3.1 depletion was confirmed by a 4.8-fold decrease in the mRNA level (Figure 2). In addition, the native expression of NKX3.1 in LNCaP cells was supplemented with ectopic NKX3.1 expression, and its role in oxidative regulation was investigated. Ectopic NKX3.1 expression significantly increased the expression of GPX2 (3.7 to 5.1) and SOD2 (3.8 to 5.9) upon CM exposure (Figure 3A). These data suggested that the restored NKX3.1 expression back to its normal levels enhanced the antioxidant response in the inflammatory microenvironment. Moreover, the intracellular ROS level was examined using the DCFH assay following the treatment of NKX3.1-overexpressing LNCaP cells with H_2_O_2_ (50 and 100 μM). Upon H_2_O_2_ treatment, a significant reduction in the ROS level was observed not only in control cells with basal NKX3.1 expression but also in cells with ectopic NKX3.1 expression (Figure 3B). The data demonstrate that LNCaP cells require NKX3.1 expression for regulation of the antioxidant response during inflammation in the prostate.

### SIRT1 leads to oxidative stress resistance in the inflammatory microenvironment

The accumulation of damage to DNA, proteins and lipids is characterized by an increase in intracellular oxidative stress levels due to a progressive decrease of ROS scavenging [18,20]. Several lines of evidence indicate that the loss of oxidative tolerance is age dependent and associated with DNA damage as well as metabolic deregulation [21]. The SIRT1-mediated increase in oxidative stress tolerance and the concurrent activation of the p53-mediated DNA damage response were correlated with an extended lifespan in mouse models. A decrease in p53 activity reduces the essential role of p53 in tumor prevention in older animals [22]. Thus, a dramatic increase in the frequency of cancer provides a likely explanation for the correlation between tumorigenesis and the accumulation of DNA mutations [23,24] that might be related to a decreased stress tolerance and increased genetic heterogeneity during multiple inflammatory exposures over a lifetime.

**Figure 4:**
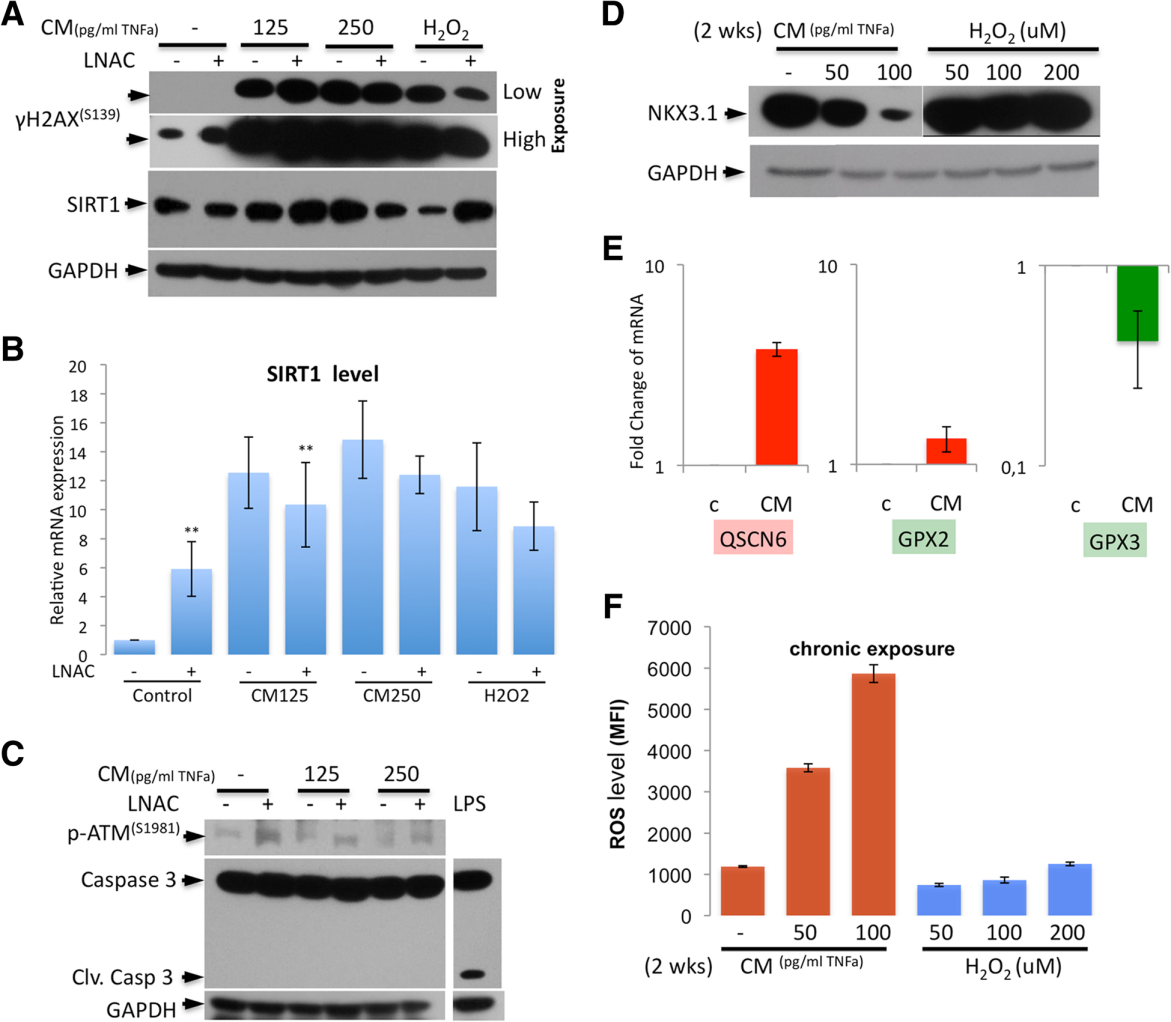
**Inflammation results in sustained oxidative damage to DNA. A**. The levels of the DNA damage marker γ-H2AX^(S139)^ and the metabolic regulator SIRT1 are remarkably affected by treatment with CM and H_2_O_2_ in the absence of LNAC. **B**. SIRT1 mRNA expression in LNCaP cells is altered by treatment with CM (including 125 and 250 pg/ml of TNFα) and 250 μM H_2_O_2_ with and without 10 mM LNAC. **C**. Caspase 3 and p-ATM^(S1981)^ levels remain lower in the treated cells in comparison to controls, indicating that the cells failed to activate apoptosis and the DNA damage response upon oxidative DNA damage. LPS (10 ng/ml) treatment was used as positive control for caspase-3 cleavage and activation. **D**. LNCaP cells were exposed to CM (including 50 and 100 pg/ml of TNFα) and H_2_O_2_ (50, 100 and 200 μM) for 2 weeks. Chronic exposure to CM, but not H_2_O_2_, results in the loss of NKX3.1. **E**. Similar to the acute treatments (24 h), chronic CM exposure (100 pg/ml of TNFα) also results in increases in QSCN6 and GPX2 expression but a decrease in GPX3 expression in LNCaP cells. Red bars represent upregulation, green bars represent downregulation. **F**. Chronic CM exposure, but not treatment with H_2_O_2_, results in an increase in the intracellular ROS level; chronic exposure to H_2_O_2_ does not affect the level of ROS in LNCaP cells. The ROS level was measured using a DCFH-DA assay with four replicates.

p53 is found to be completely lost or mutated at a high frequency in advanced prostate cancer [25], but many other tumor suppressors also contribute to the regulation of cell proliferation and apoptosis. An increase in the stability of p53 by hyperacetylation via several acetylases activates p53 to trigger apoptosis and cell cycle arrest. Conversely, the deacetylation of p53 induced by sustained SIRT1 expression enhances the destruction of p53 through ubiquitin-mediated proteasomal degradation [26]. This work suggests that the activation of SIRT1 (via decreased metabolic events) unexpectedly increases the genetic heterogeneity in the inflammatory microenvironment, where the DNA damage response may not be activated. Because high expression of SIRT1 is a common and relevant pathologic event in prostate cancer [27], we examined DNA damage, the subsequent activation of SIRT1 and the DNA damage response. We observed that oxidative stress strongly increased the γ-H2AX^(S139)^ levels (a hallmark of DNA damage) upon treatment with CM and H_2_O_2_, concurrent to the increase in SIRT1 expression/stability upon CM treatment, although the γ-H2AX^(S139)^ levels were not increased upon treatment with H_2_O_2_. However, the antioxidant (LNAC) restored SIRT1 expression to its basal level in cells exposed to high concentrations of CM (Figure 4A). Because the CM-induced DNA damage cannot be restored by LNAC, the γ-H2AX^(S139)^ level remained high upon CM treatment and was partially restored upon H_2_O_2_ treatment. CM treatment results in a significant amount of DNA damage, suggesting that the level of DNA damage is high in the inflammatory microenvironment but not in more highly oxidative conditions, in which the putative responsive pathways remain to be elucidated. On the other hand, because the relative SIRT1 mRNA level (Figure 4B) correlated with the SIRT1 protein level, we hypothesized that SIRT1 is upregulated under inflammatory conditions and that additional control mechanisms affect protein stability. These data suggest that SIRT1 is an important metabolic regulator that senses the metabolic rate and oxidative level under oxidative stress tolerance conditions.

Furthermore, because the SIRT1 level was increased in the cells treated with CM, we elucidated the DNA damage response activation by examining the p-ATM^(S1981)^ level. Interestingly, we found that ATM^(S1981)^ phosphorylation was not significantly increased upon CM treatment. LNAC only induced p-ATM^(S1981)^ in control cells and not after CM exposure, suggesting that the reduced oxidative stress levels might trigger a cell cycle progression and DNA damage response, presumably via SIRT1-mediated p53 and NBS1 activations respectively. However, this hypothesis requires further investigation. We also examined whether apoptosis was induced in response to DNA damage, and found that the ATM-mediated response upon CM exposure did not generate caspase-3 cleavage in LNCaP cells, either with or without LNAC treatment (Figure 4B). These data demonstrate that apoptosis was not activated, whereas the cells continued to proliferate with damaged DNA, resulting in genetic heterogeneity. However, this proliferation was suppressed by the metabolic activity sensor and NAD^+^-dependent deacetylase SIRT1, which slows down the cell cycle through the deacetylation of functional p53 to allow time for DNA damage repair. This process requires the presence of functional NKX3.1 in prostate cells [28,29]. This mechanism might result in oxidative stress tolerance and is commonly observed in cancer progression.

### Loss of NKX3.1 under conditions of chronic inflammation leads to an abrogated antioxidant response

Chronic inflammation with sustained oxidative stress is well known to promote carcinogenesis [2]. To mimic chronic inflammation *in vitro*, we fed LNCaP cells with CM (50 and 100 pg/ml of TNFα) or normal media or H_2_O_2_ (50, 100 and 200 μM) for a 2-week period. First, we analyzed the proteasomal degradation of NKX3.1, which correlated with exposure to increasing concentrations of CM (Figure 4C) but not with H_2_O_2_ treatments, confirming that cytokines are the major cause of NKX3.1 degradation. Secondly, we investigated the expression of antioxidant genes and detected the upregulation of the pro-oxidant QSCN6 (3.8-fold), marginal upregulation of the antioxidant GPX2 (increase 1.3-fold) and downregulation of GPX3 (2.5-fold) after CM (100 pg/ml of TNFα) treatment (Figure 4D). These alterations may be due to the chronic exposure to inflammatory cytokines, which might be sufficient to maintain cell survival. Surprisingly, treatment of cells with H_2_O_2_ did not result in significant changes in the expression of antioxidant response factors (data not shown). We also measured the intracellular ROS levels after the chronic treatments to gain insight into the changes in the oxidative conditions within the cells. Chronic CM exposure dose dependently correlated with an increase in the ROS level, but chronic H_2_O_2_ exposure maintained the ROS level close to the basal concentration (Figure 4E). These data suggest that inflammatory cytokine release is the major factor underlying the deregulated antioxidant response and sustained oxidative damage in prostate cells. Additionally, we found that NKX3.1 loss caused by CM exposure inversely correlated with an increased ROS level. When the NKX3.1 level remained stable after H_2_O_2_ exposure, there was no change in the ROS level. Thus, these data suggest that the increased ROS concentration after chronic CM exposure might be a consequence of NKX3.1 loss.

### NKX3.1 suppresses the proliferation enhanced by the inflammatory microenvironment

**Figure 5:**
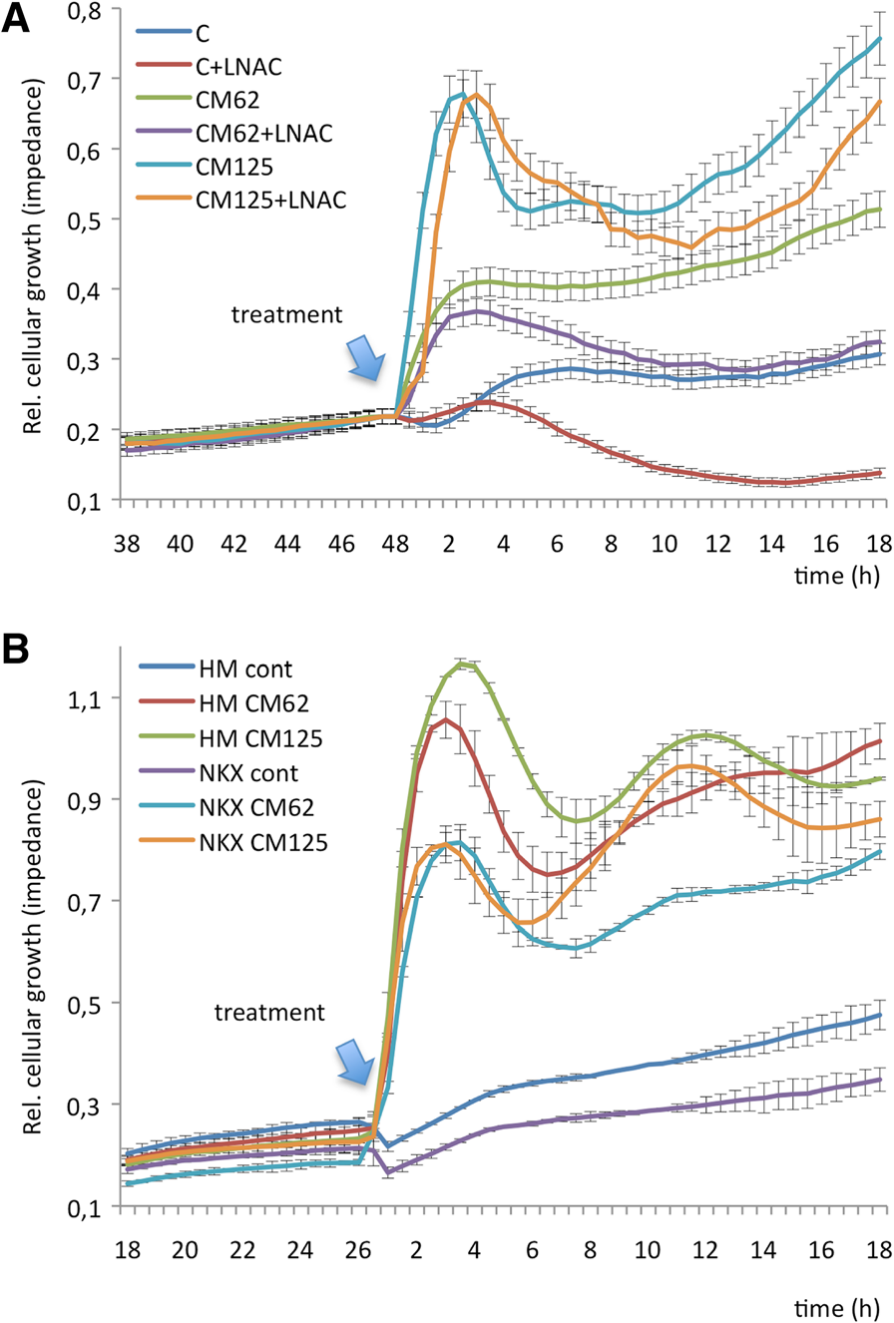
**ROS-mediated changes are partially restored upon NKX3.1 expression or LNAC treatment. A**. LNAC treatment and **B**. NKX3.1 overexpression remarkably suppresses cellular proliferation when LNCaP cells are treated with CM (including 62 and 125 pg/ml of TNFα). LNAC (10 mM) or NKX3.1 overexpression restores the CM-mediated suppression in cell proliferation. HM-NKX3.1 was transfected into the cells 24 h before CM treatment. HM: Hismax control vector, NKX: HM-NKX3.1 transfection. The Xcelligence real-time cell proliferation assay system was used to evaluate cell proliferation. The blue arrows indicate when the treatments were performed.

To investigate the influence of NKX3.1 loss on cell survival, we examined cellular proliferation. In a real-time setting for 18 h, we observed that the rate of LNCaP cell proliferation increased after (62 and 125 pg/ml of TNFα including) CM treatment. The proliferation rate was suppressed with either LNAC treatment (Figure 5A) or when NKX3.1 was expressed ectopically (Figure 5B). These data demonstrate that NKX3.1 deregulates cell proliferation induced by the inflammatory microenvironment by functioning similarly to LNAC in the response to oxidative stress.

## Conclusions

**Figure 6:**
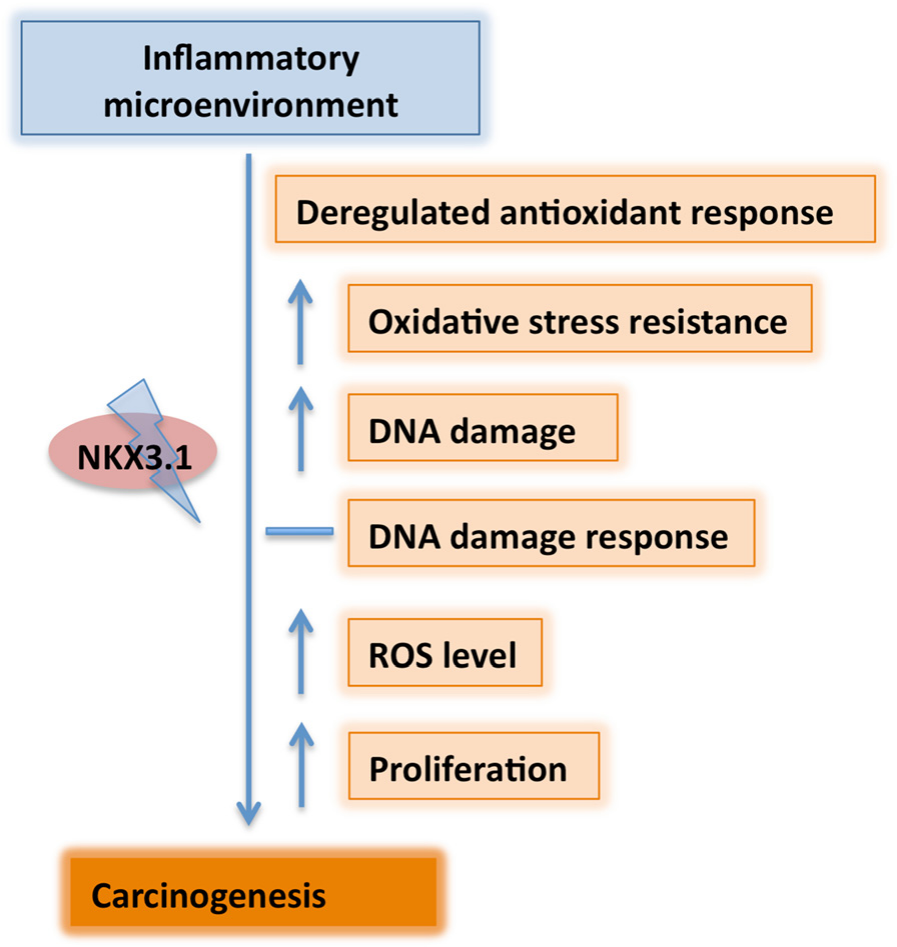
**Scheme representing the mechanism of cancer development as a result of the loss of the NKX3.1 protein in inflammation.**

Overall, the inflammatory microenvironment is the major factor leading to sustained DNA damage in LNCaP cells, and the loss of NKX3.1 is an important contributing event. Therefore, the use of antioxidants might have a limited, but important ability to suppress the associated tumorigenic alterations, particularly in prostatic inflammation related cancer development. Upon chronic exposure of LNCaP cells to CM to mimic inflammation-like conditions, the loss of NKX3.1 leads to the deregulation of oxidative stress scavengers and contributes to increased DNA damage, eventually the prostate tumor progression (Figure 6). The inflammatory microenvironment model proposed here represents a novel approach for investigating the molecular and cellular alterations in cells *in vitro*, closely resembling animal studies [19].
